# Measuring Integrated Information: Comparison of Candidate Measures in Theory and Simulation

**DOI:** 10.3390/e21010017

**Published:** 2018-12-25

**Authors:** Pedro A.M. Mediano, Anil K. Seth, Adam B. Barrett

**Affiliations:** 1 Department of Computing, Imperial College, London SW7 2RH, UK; 2 Sackler Centre for Consciousness Science and Department of Informatics, University of Sussex, Brighton BN1 9RH, UK

**Keywords:** integrated information theory, computational neuroscience, complexity, consciousness

## Abstract

Integrated Information Theory (IIT) is a prominent theory of consciousness that has at its centre measures that quantify the extent to which a system generates more information than the sum of its parts. While several candidate measures of integrated information (“Φ”) now exist, little is known about how they compare, especially in terms of their behaviour on non-trivial network models. In this article, we provide clear and intuitive descriptions of six distinct candidate measures. We then explore the properties of each of these measures in simulation on networks consisting of eight interacting nodes, animated with Gaussian linear autoregressive dynamics. We find a striking diversity in the behaviour of these measures—no two measures show consistent agreement across all analyses. A subset of the measures appears to reflect some form of dynamical complexity, in the sense of simultaneous segregation and integration between system components. Our results help guide the operationalisation of IIT and advance the development of measures of integrated information and dynamical complexity that may have more general applicability.

## 1. Introduction

Measures of integrated information seek to quantify the extent to which a whole system generates more information than the sum of its parts as it transitions between states. In biological systems, integrated information could underpin cognitive and behavioural flexibility, and even consciousness. More generally, integrated information measures have the potential to capture the dynamical complexity of any many body system, and hence to aid with understanding and characterising complex systems [1]. Since the concept of integrated information can be operationalised in many different ways, a whole range of distinct integrated information measures have come into being in the literature [2,3,4,5]. Several of them are beginning to see application to empirical data [6], or to large-scale simulations [7,8], yet a systematic comparison of the behaviour of the various measures on non-trivial network models has not previously been performed.

This paper has two goals: first, to provide a unified source of explanation of the principles and practicalities of a class of prominent candidate measures of integrated information; second, to examine the behaviour of candidate measures on non-trivial network models, in order to shed light on their comparative practical utility.

The class of integrated information measures we consider could be called *dynamical*, or *empirical*, integrated information measures. Following Barrett and Seth [2], they quantify the information that the current state contains about a past state (for the information integrated over time window τ, the past state to be considered is that at time τ from the present), measured using the empirical, or spontaneous, distribution for *a priori* uncertainty about the past state. These measures are well-defined for any stochastic system (with a well-defined Lebesgue measure across the states) and have the advantage that they can be estimated for real data using empirical distributions if stationarity can be assumed. By contrast, the integrated information measures introduced in especially more recent versions of the Integrated Information Theory of consciousness (IIT) are applicable only to discrete Markovian systems [9]. These measures, which could be called *causal* integrated information measures, compute the information generated when the system transitions to one particular state out of a repertoire of possible states; the *a priori* distribution for the past state is taken to be maximum entropy, so that the measures reflect every possible thing that the system could do.

Related to integrated information is the notion of dynamical complexity, which has been variously defined [10,11]. Sometimes dynamical complexity is considered as the whole being somehow greater than the sum of parts, and sometimes as a system showing a balance between two competing tendencies, namely

- **integration**, i.e., the system behaves as one; and
- **segregation**, i.e., the parts of the system behave independently.

The notion of dynamical complexity has further been described as a balance between order and disorder, or between chaos and synchrony, and has been related to criticality and metastability [7]. Similarly, it has been argued that a necessary feature of complexity measures is to peak in systems that exhibit a mixture between low and high correlation [11]. Many quantitative measures of dynamical complexity have been proposed, but a theoretically-principled, one-size-fits-all measure remains elusive. In exploring the behaviour of each of the integrated information measures, we consider to what extent they reflect integration, segregation, and a balanced degree of correlation.

In short, measures of integrated information and dynamical complexity share the notion of tracking the extent to which the whole is more than the sum of the parts. While early versions of IIT explicitly linked integrated information with dynamical complexity [10], later versions have focused on a “causal” definition of integrated information which has more to do with the “irreducibility of mechanisms” than the co-existence of integration and segregation in a system’s dynamics [9]. Here, we focus on the earlier, dynamical/empirical conceptions of integrated information because (i) they are more readily applicable to empirical time-series; (ii) they remain conceptually powerful in theories of consciousness, and (iii) they promise general applicability to many other questions in neuroscience and beyond, in which part-whole relations are of interest.

We consider five distinct and prominent measures of dynamical integrated information: whole-minus-sum integrated information Φ [12]; integrated stochastic interaction $\tilde{\Phi }$ [2]; integrated synergy ψ [3]; decoder-based integrated information $\Phi^{*}$ [5]; and geometric integrated information $\Phi_{G}$ [4]. We also consider, for comparison, the measure causal density (CD) [13,14], which can be considered as the sum of independent information transfers in the system (without reference to a minimum information partition). This measure has previously been discussed in conjunction with integrated information and dynamical complexity measures [13,15]. For a comparison of related complexity measures, see Reference [16].

All of the measures have the potential to behave in ways which are not obvious a priori, and in a manner difficult to express analytically. While some simulations of some of the measures (Φ, $\tilde{\Phi }$, CD) on networks have been performed [2,13], and some analytical understanding has been achieved for Φ and $\tilde{\Phi }$ [5,17], other measures ($\Phi^{*}$, $\Phi_{G}$) have not previously been computed on any model consisting of more than two components. This paper provides a comparison of the full suite of measures on non-trivial network models. We consider eight-node networks with a range of different architectures, animated with basic noisy vector autoregressive dynamics. We examine how each measure is affected by network topology, coupling strength and noise correlation, as well as its relation with global correlation (a simple dynamical control). Based on these comparisons, we discuss the extent to which each measure captures the co-existence of integration and segregation central to the concept of dynamical complexity.

After covering the necessary preliminaries, we set out the intuition behind the measures, and summarise the mathematics behind the definition of each measure. Next, we present the simulations and their results, and conclude with a discussion of the implications for IIT. In the Appendices, we derive new formulae for computing the decoder-based integrated information $\Phi^{*}$ for Gaussian systems, correcting the previous formulae in Reference [5], and present further derivations of mathematical properties of the measures.

## 2. Methods

### 2.1. Notation, Convention and Preliminaries

In this section, we review the fundamental concepts needed to define and discuss the candidate measures of integrated information. For a more comprehensive introduction, see Reference [18]. In general, we will denote random variables with uppercase letters (e.g., *X*, *Y*) and particular instantiations with the corresponding lowercase letters (e.g., *x*, *y*). Variables can be either continuous or discrete, and we assume that continuous variables can take any value in $R^{n}$ and that a discrete variable *X* can take any value in the finite set $\Omega_{X}$. Whenever there is a sum involving a discrete variable *X*, we assume the sum runs for all possible values of *X* (i.e., the whole $\Omega_{X}$). A partition $P=\{M^{1},M^{2},\cdots ,M^{r}\}$ divides the elements of system *X* into *r* non-overlapping, non-empty sub-systems (or parts), such that $X=M^{1}⋃M^{2}⋃\cdots ⋃M^{r}$ and $M^{i}⋂M^{j}=\emptyset$, for any i,j. We denote each variable in *X* as $X^{i}$, and the total number of variables in *X* as *n*. When dealing with time series, time will be indexed with a subscript, e.g., $X_{t}$.

*Entropy H* quantifies the uncertainty associated with random variable *X*—i.e., the higher H(X) the harder it is to make predictions about *X*—and is defined as

(1) $$\begin{array}{c} H\left(X\right)=:-\sum_{x}p\left(x\right)\log p\left(x\right). \end{array}$$

In many scenarios, a discrete set of states is insufficient to represent a process or time series. This is the case, for example, with brain recordings, which come in real-valued time series and with no a priori discretisation scheme. In these cases, using a continuous variable X∈R, we can similarly define the *differential entropy*,

(2) $$\begin{array}{c} H[p]=:-\int p(x)\log p(x)dx. \end{array}$$

However, differential entropy is not as interpretable and well-behaved as its discrete-variable counterpart. For example, differential entropy is not invariant to rescaling or other transformations on *X*. Moreover, it is only defined if *X* has a density with respect to the Lebesgue measure dx; this assumption will be upheld throughout this paper. We can also define the *conditional* and *joint* entropies as

(3) $$\begin{array}{c} \begin{array}{cc} H(X\;|\;Y) & =:\sum_{y}p\left(y\right)H\left(X\;|\;Y=y\right)\\ & =-\sum_{y}p\left(y\right)\sum_{x}p\left(x\;|\;y\right)\log p\left(x\;|\;y\right), \end{array} \end{array}$$

(4) $$\begin{array}{c} H\left(X,Y\right)=:-\sum_{x,y}p\left(x,y\right)\log p\left(x,y\right), \end{array}$$

respectively. Conditional and joint entropies can be analogously defined for continuous variables by appropriately replacing sums with integrals.

The Kullback–Leibler (KL) divergence quantifies the dissimilarity between two probability distributions *p* and *q*:

(5) $$D_{KL}\left(p∥q\right)=:\sum_{x}p\left(x\right)\log\frac{p(x)}{q(x)}.$$

The KL divergence represents a notion of (non-symmetric) distance between two probability distributions. It plays an important role in information geometry, which deals with the geometric structure of manifolds of probability distributions.

Finally, mutual information *I* quantifies the interdependence between two random variables *X* and *Y*. It is the KL divergence between the full joint distribution and the product of marginals, but it can also be expressed as the average reduction in uncertainty about *X* when *Y* is given:

(6) $$\begin{array}{c} \begin{array}{cc} I(X;Y) & =:D_{KL}\left(p(X,Y)\;∥\;p(X)p(Y)\right)\\ & =H(X)+H(Y)-H(X,Y)\\ & =H(X)-H(X|Y). \end{array} \end{array}$$

Mutual information is symmetric in the two arguments *X* and *Y*. We make use of the following properties of mutual information:

1. I(X;Y)=I(Y;X),
2. I(X;Y)≥0, and
3. I(f(X);g(Y))=I(X;Y) for any injective functions f,g.

We highlight one implication of property 3: *I* is upper-bounded by the entropy of both *X* and *Y*. This means that the entropy H(X) of a random variable *X* is the maximum amount of information *X* can have about any other variable *Y* (or another variable *Y* can have about *X*).

Mutual information is defined analogously for continuous variables and, unlike differential entropy, it retains its interpretability in the continuous case [19]. Furthermore, one can track how much information a system preserves during its temporal evolution by computing the time-delayed mutual information (TDMI) $I(X_{t};X_{t-\tau })$.

Next, we introduce notation and several useful identities to handle Gaussian variables. Given an *n*-dimensional real-valued system *X*, we denote its covariance matrix as $\Sigma {\left(X\right)}_{ij}=:\mathrm{cov}\left(X^{i},X^{j}\right)$. Similarly, cross-covariance matrices are denoted as $\Sigma {\left(X,Y\right)}_{ij}=:\mathrm{cov}\left(X^{i},Y^{j}\right)$. We will make use of the conditional (or partial) covariance formula,

(7) $$\begin{array}{c} \Sigma \left(X|Y\right)=:\Sigma \left(X\right)-\Sigma \left(X,Y\right)\Sigma {\left(Y\right)}^{-1}\Sigma \left(Y,X\right). \end{array}$$

For Gaussian variables,

(8) $$\begin{array}{cc} H(X) & =\frac{1}{2}\log\left(\det\Sigma \left(X\right)\right)+\frac{1}{2}n\log\left(2\pi e\right)\;, \end{array}$$

(9) $$\begin{array}{cc} H(X|Y=y) & =\frac{1}{2}\log\left(\det\Sigma \left(X|Y\right)\right)+\frac{1}{2}n\log\left(2\pi e\right)\;,\;\;\;\forall y\;, \end{array}$$

(10) $$\begin{array}{cc} I(X;Y) & =\frac{1}{2}\log\left(\frac{\det\Sigma (X)}{\det\Sigma (X|Y)}\right).\; \end{array}$$

All systems we deal with in this article are stationary and ergodic, so throughout the paper $\Sigma \left(X_{t}\right)=\Sigma \left(X_{t-\tau }\right)$ for any τ.

### 2.2. Integrated Information Measures

#### 2.2.1. Overview

In this section, we review the theoretical underpinnings and practical considerations of several proposed measures of integrated information, and in particular how they relate to intuitions about segregation, integration and complexity. These measures are:

- Whole-minus-sum integrated information, Φ;
- Integrated stochastic interaction, $\tilde{\Phi }$;
- Integrated synergy, ψ;
- Decoder-based integrated information, $\Phi^{*}$;
- Geometric integrated information, $\Phi_{G}$; and
- Causal density, CD.

All of these measures (besides CD) have been inspired by the measure proposed by Balduzzi and Tononi in [12], which we call $\Phi_{2008}$. $\Phi_{2008}$ was based on the information the current state contains about a hypothetical maximum entropy past state. In practice, this results in measures that are applicable only to discrete Markovian systems [2]. For broader applicability, it is more practical to build measures based on the ongoing spontaneous information dynamics—that is, based on $p(X_{t},X_{t-\tau })$ without applying a perturbation to the system. Measures are then well-defined for any stochastic system (with a well-defined Lebesgue measure across the states), and can be estimated for real data using empirical distributions if stationarity can be assumed. All of the measures we consider in this paper are based on a system’s spontaneous information dynamics.

Table 1 contains a brief description of each measure and a reference to the original publication that introduced it [20]. We refer the reader to the original publications for more detailed descriptions of each measure. Table 2 contains a summary of properties of the measures considered, proven for the case in which the system is ergodic and stationary, and the spontaneous distribution is used.

#### 2.2.2. Minimum Information Partition

Key to all measures of integrated information is the notion of splitting or partitioning the system to quantify the effect of such split on the system as a whole. In that spirit, integrated information measures are defined through some measure of *effective information*, which operationalises the concept of “information *beyond* a partition” P. This typically involves splitting the system according to P and computing some form of information loss, via (for example) mutual information (Φ), conditional entropy ($\tilde{\Phi }$), or decoding accuracy ($\Phi^{*}$) (see Table 1). Integrated information is then the effective information with respect to the partition that identifies the “weakest link” in the system, i.e., the partition for which the parts are least integrated. Formally, integrated information is the effective information beyond the *minimum information partition* (MIP), which, given an effective information measure f[X;τ,P], is defined as

(11) $$\begin{array}{c} P_{\mathrm{MIP}}=\arg_{P}\min\frac{f[X;\tau ,P]}{K(P)}, \end{array}$$

where K(P) is a normalisation coefficient. In other words, the MIP is the partition across which the (normalised) effective information is minimum, and integrated information is the (unnormalised) effective information beyond the MIP. The purpose of the normalisation coefficient is to avoid biasing the minimisation towards unbalanced bipartitions (recall that the extent of information sharing between parts is bounded by the entropy of the smaller part). Balduzzi and Tononi [12] suggest the form

(12) $$\begin{array}{c} K\left(P\right)=\left(r-1\right)\min_{k}H\left(M_{t}^{k}\right). \end{array}$$

However, not all contributions to IIT have followed Balduzzi and Tononi’s treatment of the MIP. Of the measures listed above, Φ and $\tilde{\Phi }$ share this partition scheme, ψ defines the MIP through an *unnormalised* effective information, and $\Phi^{*}$, $\Phi_{G}$ and CD are defined via the atomic partition without any reference to the MIP. These differences are a confounding factor when it comes to comparing measures—it becomes difficult to ascertain whether differences in behaviour of various measures are due to their definitions of effective information, to their normalisation factor (or lack thereof), or to their partition schemes. We return to this topic in the Discussion section below.

In the following, we present all measures as they were introduced in their original papers (see Table 1), although it is trivial to combine different effective information measures with different partition optimisation schemes. However, all results presented here are calculated by minimising each unnormalised effective information measure over even-sized bipartitions—i.e., bipartitions in which both parts have the same number of components. This is to avoid conflating the effect of the partition scan method with the effect of the integrated information measure itself.

#### 2.2.3. Whole-Minus-Sum Integrated Information Φ

We next turn to the different measures of integrated information. As highlighted above, a primary difference among them is how they define the effective information beyond a given partition. Since most measures were inspired by Balduzzi and Tononi’s $\Phi_{2008}$, we start there.

For $\Phi_{2008}$, the effective information $\varphi_{2008}$ is written as (following notation from [21]) the KL divergence between $p_{c}\left(X_{0}|X_{1}=x\right)$ and $\Pi_{k}p_{c}\left(M_{0}^{k}|M_{1}^{k}=m^{k}\right)$, where $p_{c}\left(X_{0}|X_{1}=x\right)$ (and analogously $p_{c}\left(M_{0}^{k}|M_{1}^{k}=m^{k}\right)$) is the conditional distribution for $X_{0}$ given $X_{1}=x$ under the perturbation at time 0 into all states with equal probability—i.e., given that the joint distribution is given by $p_{\mathrm{ce}}\left(X_{0},X_{1}\right)=:p\left(X_{1}|X_{0}\right)p_{u}\left(X_{0}\right)$, where $p_{u}$ is the uniform (maximum entropy) distribution [22].

Averaging $\varphi_{2008}$ over all states *x*, the result can be expressed as either

(13) $$\begin{array}{c} I\left(X_{0};X_{1}\right)-\sum_{k=1}^{r}I\left(M_{0}^{k};M_{1}^{k}\right), \end{array}$$

or

(14) $$\begin{array}{c} -H\left(X_{0}|X_{1}\right)+\sum_{k=1}^{r}H\left(M_{0}^{k}|M_{1}^{k}\right). \end{array}$$

These two expressions are equivalent under the uniform perturbation, since they differ only by a factor that vanishes if $p(X_{0})$ is the uniform distribution. However, they are *not* equivalent if the spontaneous distribution of the system is used instead—i.e., if $p(X_{t-\tau },X_{t})$ is used instead of $p_{\mathrm{ce}}\left(X_{0},X_{1}\right)$. This means that, for application to spontaneous dynamics (i.e., without perturbation), we have two alternatives that give rise to two measures that are both equally valid analogs of $\Phi_{2008}$.

We call the first alternative whole-minus-sum integrated information Φ ($\Phi_{E}$ in [2]). The effective information φ is defined as the difference in time-delayed mutual information between the whole system and the parts. The effective information of the system beyond a certain partition P is

(15) $$\begin{array}{c} \varphi \left[X;\tau ,P\right]=:I\left(X_{t-\tau };X_{t}\right)-\sum_{k=1}^{r}I\left(M_{t-\tau }^{k};M_{t}^{k}\right)\;. \end{array}$$

We can interpret $I(X_{t-\tau };X_{t})$ as how good the system is at predicting its own future or decoding its own past (which are equivalent because mutual information is symmetric). Then, φ here can be seen as the loss in predictive power incurred by splitting the system according to P. The details of the calculation of Φ (and the MIP) are shown in Box 1.

Φ is often regarded as a poor measure of integrated information because it can be negative, as established in known analytical results [4,5]. This is indeed conceptually awkward if Φ is seen as an absolute measure of integration between the parts of a system, though it is a reasonable property if Φ is interpreted as a “net synergy” measure [23]—quantifying to what extent the parts have shared or complementary information about the future state. That is, if Φ>0, we infer that the whole is better than the parts at predicting the future (i.e., Φ>0 is a sufficient condition), but a negative or zero Φ does not imply the opposite. Therefore, from an IIT perspective, a negative Φ can lead to the understandably confusing interpretation of a system having “negative integration”, but, through a different lens (net synergy), it can be more easily interpreted as overall redundancy in the evolution of the system. See the section on integrated synergy ψ and Reference [23] for further discussion on whole-minus-sum measures.

**Box 1. Calculating whole-minus-sum integrated information Φ.**

(16a) $$\begin{array}{c} \Phi \left[X;\tau \right]=\varphi \left[X;\tau ,B^{\mathrm{MIB}}\right] \end{array}$$

(16b) $$\begin{array}{c} B^{\mathrm{MIB}}=\arg_{B}\min\frac{\varphi [X;\tau ,B]}{K(B)} \end{array}$$

(16c) $$\begin{array}{c} \varphi \left[X;\tau ,B\right]=I\left(X_{t-\tau };X_{t}\right)-\sum_{k=1}^{2}I\left(M_{t-\tau }^{k};M_{t}^{k}\right) \end{array}$$

(16d) $$\begin{array}{c} K\left(B\right)=\min\left\{H\left(M_{t}^{1}\right),H\left(M_{t}^{2}\right)\right\} \end{array}$$

1. For **discrete variables**:
  $$\begin{array}{c} I\left(X_{t-\tau };X_{t}\right)=\sum_{x,x^{\prime }}p\left(X_{t-\tau }=x,X_{t}=x^{\prime }\right)\log\left(\frac{p(X_{t-\tau }=x,X_{t}=x^{\prime })}{p\left(X_{t-\tau }=x\right)\;p\left(X_{t}=x^{\prime }\right)}\right) \end{array}$$
2. For **continuous, linear-Gaussian variables**:
  $$\begin{array}{c} I\left(X_{t-\tau };X_{t}\right)=\frac{1}{2}\log\left(\frac{\det\Sigma (X_{t})}{\det\Sigma (X_{t}\;|\;X_{t-\tau })}\right) \end{array}$$
3. For **continuous variables** with an arbitrary distribution, we must resort to the nearest-neighbour methods introduced by [24]. See reference for details.

#### 2.2.4. Integrated Stochastic Interaction $\tilde{\Phi }$

We next consider the second alternative for $\Phi_{2008}$ for spontaneous information dynamics: integrated stochastic interaction $\tilde{\Phi }$. Also introduced in Barrett and Seth [2], this measure embodies similar concepts as Φ, with the main difference being that $\tilde{\Phi }$ utilises a definition of effective information in terms of an *increase in uncertainty* instead of in terms of a *loss of information*.

$\tilde{\Phi }$ is based on *stochastic interaction* $\tilde{\varphi }$, introduced by Ay [25]. Akin to Equation (15), we define stochastic interaction beyond partition P as

(17) $$\begin{array}{c} \tilde{\varphi }\left[X;\tau ,P\right]=:\sum_{k=1}^{r}H\left(M_{t-\tau }^{k}\;|\;M_{t}^{k}\right)-H\left(X_{t-\tau }\;|\;X_{t}\right). \end{array}$$

Stochastic interaction quantifies to what extent uncertainty about the past is increased when the system is split in parts, compared to considering the system as a whole. The details of the calculation of $\tilde{\Phi }$ are similar to those of Φ and are described in Box 2.

The most notable advantage of $\tilde{\Phi }$ over Φ as a measure of integrated information is that $\tilde{\Phi }$ is guaranteed to be non-negative. In fact, as mentioned above, φ and $\tilde{\varphi }$ are related through the equation

(18) $$\begin{array}{c} \tilde{\varphi }\left[X;\tau ,P\right]=\varphi \left[X;\tau ,P\right]+I\left(M_{t}^{1};M_{t}^{2};\ldots ;M_{t}^{r}\right), \end{array}$$

where

(19) $$\begin{array}{c} I\left(M_{t}^{1};M_{t}^{2};\ldots ;M_{t}^{r}\right)=\sum_{k=1}^{r}H\left(M_{t}^{k}\right)-H\left(X_{t}\right). \end{array}$$

This measure is also linked to *information destruction*, as presented by Wiesner et al. [26]. The quantity $H(X_{t-\tau }|X_{t})$ measures the amount of irreversibly destroyed information, since $H(X_{t-\tau }|X_{t})>0$ indicates that more than one possible past trajectory of the system converged on the same present state, making the system irreversible and indicating a loss of information about the past states. From this perspective, $\tilde{\varphi }$ can be understood as the difference between the information that is considered destroyed when the system is observed as a whole, or split into parts. Note, however, that this measure is time-symmetric when applied to a stationary system; for stationary systems, total instantaneous entropy does not change with time. Furthermore, we know that $\tilde{\Phi }$ can exceed TDMI in some cases and that it quantifies a mixture of both causal and simultaneous influences [5].

$$\mathrm{Box}\;2\;.\;\mathrm{Calculating}\;\mathrm{integrated}\;\mathrm{stochastic}\;\mathrm{interaction}\;\tilde{\Phi }.$$

(20a) $$\begin{array}{c} \tilde{\Phi }\left[X;\tau \right]=\tilde{\varphi }\left[X;\tau ,B^{\mathrm{MIB}}\right] \end{array}$$

(20b) $$\begin{array}{c} B^{\mathrm{MIB}}=\arg_{B}\min\frac{\tilde{\varphi }\left[X;\tau ,B\right]}{K(B)} \end{array}$$

(20c) $$\begin{array}{c} \tilde{\varphi }\left[X;\tau ,B\right]=\sum_{k=1}^{2}H\left(M_{t-\tau }^{k}\;|\;M_{t}^{k}\right)-H\left(X_{t-\tau }\;|\;X_{t}\right) \end{array}$$

(20d) $$\begin{array}{c} K\left(B\right)=\min\left\{H\left(M_{t}^{1}\right),H\left(M_{t}^{2}\right)\right\} \end{array}$$

1. For **discrete variables**:
  $$\begin{array}{c} H\left(X_{t-\tau }\;|\;X_{t}\right)=-\sum_{x,x^{\prime }}p\left(X_{t-\tau }=x,X_{t}=x^{\prime }\right)\log\left(\frac{p(X_{t-\tau }=x,X_{t}=x^{\prime })}{p(X_{t}=x^{\prime })}\right) \end{array}$$
2. For **continuous, linear-Gaussian variables**:
  $$\begin{array}{c} H\left(X_{t-\tau }\;|\;X_{t}\right)=\frac{1}{2}\log\det\Sigma \left(X_{t-\tau }\;|\;X_{t}\right)+\frac{1}{2}n\log\left(2\pi e\right) \end{array}$$
3. For **continuous variables** with an arbitrary distribution, we must resort to the nearest-neighbour methods introduced by [24]. See reference for details.

#### 2.2.5. Integrated Synergy ψ

Originally designed as a “more principled” integrated information measure [3], ψ shares some features with Φ and $\tilde{\Phi }$ but is grounded in a different branch of information theory, namely the Partial Information Decomposition (PID) framework [27]. In PID, the information that two (source) variables provide about a third (target) variable is decomposed into four non-negative terms as

$$\begin{array}{c} I\left(X,Y;Z\right)=U_{X}\left(X;Z\right)+U_{Y}\left(Y;Z\right)+R\left(X,Y;Z\right)+S\left(X,Y;Z\right), \end{array}$$

where $U_{\alpha }$ is the *unique information* of source α, *R* is the *redundancy* between both sources and *S* is their *synergy*.

Integrated synergy ψ is the information that the parts provide about the future of the system that is exclusively synergistic—i.e., cannot be provided by any combination of parts independently:

(21) $$\begin{array}{c} \psi \left[X;\tau ,P\right]=:I\left(X_{t-\tau };X_{t}\right)-\max_{P}I_{\cup }\left(M_{t-\tau }^{1},M_{t-\tau }^{2},\cdots ,M_{t-\tau }^{r};X_{t}\right), \end{array}$$

where

(22) $$\begin{array}{c} I_{\cup }\left(M_{t-\tau }^{1},\ldots ,M_{t-\tau }^{r};X_{t}\right)=:\;\;\;\sum_{S\subseteq \{M^{1},\ldots ,M^{r}\}}\;\;\;{\left(-1\right)}^{|S|+1}I_{\cap }\left(S_{t-\tau }^{1},\ldots ,S_{t-\tau }^{\left|S\right|};X_{t}\right), \end{array}$$

and $I_{\cap }\left(S_{1},\ldots ,S_{\left|S\right|};Z\right)$ denotes the redundant information sources $S_{1},\ldots ,S_{\left|S\right|}$ have about target *Z*. The main problem of PID is that it is underdetermined. For example, for the case of two sources, Shannon’s information theory specifies three quantities (I(X,Y;Z), I(X;Z), I(Y;Z)), whereas PID specifies four (*S*, *R*, $U_{X}$, $U_{Y}$). Therefore, a complete operational definition of ψ requires a definition of redundancy from which to construct the partial information components [27]. In this sense, the main shortcoming of ψ, inherited from PID, is that there is no agreed consensus on a definition of redundancy [23,28].

Here, we take Griffith’s conceptual definition of ψ and we complement it with available definitions of redundancy (see Box 3). For the linear-Gaussian systems, we study here we use the minimum mutual information PID presented in [23,29]. Although we do not show any discrete examples here, for completeness, we provide complete formulae to calculate ψ for discrete variables using Griffith and Koch’s redundancy measure [30]. Note that alternatives are available for both discrete and linear-Gaussian systems [27,31,32,33,34].

**Box 3. Calculating integrated synergy *ψ*.**

(23) $$\begin{array}{c} \psi \left[X;\tau ,P\right]=I\left(X_{t-\tau };X_{t}\right)-\max_{P}I_{\cup }\left(M_{t-\tau }^{1},\ldots ,M_{t-\tau }^{r};X_{t}\right) \end{array}$$

1. For **discrete variables**: (following Griffith and Koch’s [30] PID scheme)
  $$\begin{array}{cc} I_{\cup }\left(M_{t-\tau }^{1},\ldots ,M_{t-\tau }^{r};X_{t}\right)= & \min_{q}\sum_{x,x^{\prime }}q\left(x,x^{\prime }\right)\log\left(\frac{q(x,x^{\prime })}{q\left(x\right)\;q\left(x^{\prime }\right)}\right)\\ s.t.\;\; & q\left(M_{t-\tau }^{i},X_{t}\right)=p\left(M_{t-\tau }^{i},X_{t}\right) \end{array}$$
2. For **continuous, linear-Gaussian variables**:
  $$\begin{array}{c} I_{\cup }\left(M_{t-\tau }^{1},\ldots ,M_{t-\tau }^{r};X_{t}\right)=\max_{k}I\left(M_{t-\tau }^{k};X_{t}\right) \end{array}$$
3. For **continuous variables** with an arbitrary distribution: unknown.

#### 2.2.6. Decoder-Based Integrated Information $\Phi^{*}$

Introduced by Oizumi et al. in Reference [5], decoder-based integrated information $\Phi^{*}$ takes a different approach from the previous measures. In general, $\Phi^{*}$ is given by

(24) $$\begin{array}{c} \Phi^{*}\left[X;\tau ,P\right]=:I\left(X_{t-\tau };X_{t}\right)-I^{*}\left[X;\tau ,P\right]\;, \end{array}$$

where $I^{*}$ is known as the *mismatched decoding information*, and quantifies how much information can be extracted from a variable if the receiver is using a suboptimal (or *mismatched*) decoding distribution [35,36]. This mismatched information has been used in neuroscience to quantify the contribution of neural correlations in stimulus coding [37], and can similarly be used to measure the contribution of inter-partition correlations to predictive information.

To calculate $\Phi^{*}$, we formulate a restricted model *q* in which the correlations between partitions are ignored,

(25) $$\begin{array}{c} q\left(X_{t}|X_{t-\tau }\right)=\prod_{i}p\left(M_{t}^{i}|M_{t-\tau }^{i}\right), \end{array}$$

and we calculate $I^{*}$ for the case where the sender is using the full model *p* as an encoder and the receiver is using the restricted model *q* as a decoder. The details of the calculation of $\Phi^{*}$ and $I^{*}$ are shown in Box 4. Unlike the previous measures shown in this section, $\Phi^{*}$ does not have an interpretable formulation in terms of simpler information-theoretic functionals like entropy and mutual information.

Calculating $I^{*}$ involves a one-dimensional optimisation problem, which is straightforwardly solvable if the optimised quantity, $\tilde{I}\left(\beta \right)$, has a closed form expression [35]. For systems with continuous variables, it is in general very hard to estimate $\tilde{I}\left(\beta \right)$. However, for continuous linear-Gaussian systems and for discrete systems, $\tilde{I}\left(\beta \right)$ has an analytic closed form as a function of β if the covariance or joint probability table of the system are known, respectively. In Appendix A, we derive the formulae. (Note that the version written down in Reference [5] is incorrect, although their simulations match our results; we checked results from our derived version of the formulae versus results obtained from numerical integration, and confirmed that our derived formulae are the correct ones.) Conveniently, in both the discrete and the linear-Gaussian case, $\tilde{I}\left(\beta \right)$ is concave in β (proofs in Reference [35] and in Appendix A, respectively), which makes the optimisation significantly easier.

Box 4. Calculating decoder-based integrated information Φ*.

(26a) $$\begin{array}{c} \Phi^{*}\left[X;\tau ,P\right]=I\left(X_{t-\tau };X_{t}\right)-I^{*}\left[X;\tau ,P\right] \end{array}$$

(26b) $$\begin{array}{c} I^{*}\left[X;\tau ,P\right]=\max_{\beta }\tilde{I}\left(\beta ;X,\tau ,P\right) \end{array}$$

1. For **discrete variables**:
  $$\begin{array}{c} \tilde{I}\left(\beta ;X,\tau ,P\right)=-\sum_{x^{\prime }}p\left(X_{t}=x^{\prime }\right)\log\sum_{x}p\left(X_{t-\tau }=x\right)q{\left(X_{t}=x^{\prime }|X_{t-\tau }=x\right)}^{\beta }\\ +\sum_{x,x^{\prime }}p\left(X_{t-\tau }=x,X_{t}=x^{\prime }\right)\log q{\left(X_{t}=x^{\prime }|X_{t-\tau }=x\right)}^{\beta } \end{array}$$
2. For **continuous, linear-Gaussian variables**: (see appendix for details)
  $$\begin{array}{c} \tilde{I}\left(\beta ;X,\tau ,P\right)=\frac{1}{2}\log\left(\left|Q|\right|\Sigma_{x}|\right)+\frac{1}{2}\mathrm{tr}\left(\Sigma_{x}R\right)+\beta \mathrm{tr}\left(\Pi_{x|\tilde{x}}^{-1}\Pi_{x\tilde{x}}\Pi_{x}^{-1}\Sigma_{\tilde{x}x}\right) \end{array}$$
3. For **continuous variables** with an arbitrary distribution: unknown.

#### 2.2.7. Geometric Integrated Information $\Phi_{G}$

In Reference [4], Oizumi et al. approach the notion of dynamical complexity via yet another formalism. Their approach is based on *information geometry* [38,39]. The objects of study in information geometry are spaces of families of probability distributions, considered as differentiable (smooth) manifolds. The natural metric in information geometry is the Fisher information metric, and the KL divergence provides a natural measure of (asymmetric) distance between probability distributions. Information geometry is the application of differential geometry to the relationships and structure of probability distributions.

To quantify integrated information, Oizumi et al. [4] consider the divergence between the complete model of the system under study $p(X_{t-\tau },X_{t})$ and a *restricted model* $q(X_{t-\tau },X_{t})$ in which links between the parts of the system have been severed. This is known as the *M-projection* of the system onto the manifold of restricted models $Q=\{q\;:q\left(M_{t}^{i}|X_{t-\tau }\right)=q\left(M_{t}^{i}|M_{t-\tau }^{i}\right)\}$, and

(27) $$\begin{array}{c} \Phi_{G}\left[X;\tau ,P\right]=:\min_{q\in Q}D_{KL}\left(p\left(X_{t-\tau },X_{t}\right)∥q\left(X_{t-\tau },X_{t}\right)\right). \end{array}$$

Key to this measure is that, in considering the partitioned system, it is only the connections that are cut; correlations between the parts are still allowed on the partitioned system. Although conceptually simple, $\Phi_{G}$ is very hard to calculate compared to all other measures we consider here (see Box 5). There is no known closed form solution for any system, and we can only find approximate numerical estimates for some systems. In particular, for discrete and linear-Gaussian variables, we can formulate $\Phi_{G}$ as the solution of a pure constrained multivariate optimisation problem, with the advantage that the optimisation objective is differentiable and convex [40].

**Box 5. Calculating geometric integration Φ_*G*_.**

(28a) $$\begin{array}{cc} \Phi_{G}\left[X;\tau ,P\right]= & \min_{q}D_{KL}\left(p∥q\right) \end{array}$$

(28b) $$\begin{array}{cc} s.t.\;\; & q\left(M_{t+\tau }^{i}\;|\;X_{t}\right)=q\left(M_{t+\tau }^{i}\;|\;M_{t}^{i}\right). \end{array}$$

1. For **discrete variables**: numerically optimise the objective $D_{KL}\left(p∥q\right)$ subject to the constraints
  $$\begin{array}{c} \sum_{x,x^{\prime }}q\left(X_{t-\tau }=x^{\prime },X_{t}=x\right)=1\;\mathrm{and}\;q\left(M_{t}^{i}\;|\;X_{t-\tau }\right)=q\left(M_{t}^{i}\;|\;M_{t-\tau }^{i}\right)\;\forall i. \end{array}$$
2. For **continuous, linear-Gaussian variables**: numerically optimise the objective
  $$\begin{array}{c} \Phi_{G}\left[X;\tau ,P\right]=\min_{\Sigma {\left(E\right)}^{\prime }}\frac{1}{2}\log\frac{{|\Sigma \left(E\right)}^{\prime }|}{\left|\Sigma (E)\right|}\;, \end{array}$$
  where $\Sigma \left(E\right)=\Sigma \left(X_{t}|X_{t-1}\right)$, and subject to the constraints
  $$\begin{array}{c} \Sigma {\left(E\right)}^{\prime }=\Sigma \left(E\right)+\left(A-A^{\prime }\right)\Sigma \left(X\right){\left(A-A^{\prime }\right)}^{T}\;\mathrm{and}\\ {\left(\Sigma \left(X\right)\left(A-A^{\prime }\right)\Sigma {\left(E\right)}^{\prime -1}\right)}_{ii}=0. \end{array}$$
3. For **continuous variables** with an arbitrary distribution: unknown.

#### 2.2.8. Causal Density

Causal density (CD) is somewhat distinct from the other measures considered so far, in the sense that it is a sum of information transfers rather than a direct measure of the extent to which the whole is greater than the parts. Nevertheless, we include it here because of its relevance and use in the dynamical complexity literature.

CD was originally defined in terms of Granger causality [14,41], but here we write it in terms of Transfer Entropy (TE), which provides a more general information-theoretic definition [42]. The conditional transfer entropy from *X* to *Y* conditioned on *Z* is defined as

(29) $$\begin{array}{c} {\mathrm{TE}}_{\tau }\left(X\to Y\;|\;Z\right)=:I\left(X_{t};Y_{t+\tau }\;|\;Z_{t},Y_{t}\right). \end{array}$$

With this definition of TE, we define CD as the average pairwise conditioned TE between all variables in *X*,

(30) $$\begin{array}{c} \mathrm{CD}\left[X;\tau ,P\right]=:\frac{1}{r(r-1)}\sum_{i\neq j}{\mathrm{TE}}_{\tau }\left(M^{i}\to M^{j}\;|\;M^{\left[ij\right]}\right), \end{array}$$

where $M^{\left[ij\right]}$ is the subsystem formed by all variables in *X* except for those in parts $M^{i}$ and $M^{j}$.

In a practical sense, CD has many advantages. It has been thoroughly studied in theory [43] and applied in practice, with application domains ranging from complex systems to neuroscience [44,45,46]. Furthermore, there are off-the-shelf algorithms that calculate TE in discrete and continuous systems [47]. For details of the calculation of CD, see Box 6.

Causal density is a principled measure of dynamical complexity, as it vanishes for purely segregated or purely integrated systems. In a highly segregated system, there is no information transfer at all, and, in a highly integrated system, there is no transfer from one variable to another beyond the rest of the system [13]. Furthermore, CD is non-negative and upper-bounded by the total time-delayed mutual information (proof in Appendix B), therefore satisfying what other authors consider an essential requirement for a measure of integrated information [4].

**Box 6. Calculating causal density CD.**

(31) $$\begin{array}{c} \mathrm{CD}\left[X;\tau ,P\right]=\frac{1}{r(r-1)}\sum_{i\neq j}{\mathrm{TE}}_{\tau }\left(M^{i}\to M^{j}\;|\;M^{\left[ij\right]}\right) \end{array}$$

1. For **discrete variables**:
  $$\begin{array}{c} \begin{array}{c} TE_{\tau }(X^{i}\to X^{j}\;|\;X^{\left[ij\right]})=\\ \;\;\;\sum_{x,x^{\prime }}p\left(X_{t+\tau }^{j}=x^{\prime j},X_{t}=x\right)\log\left(\frac{p\left(X_{t+\tau }^{j}=x^{\prime j}\;|\;X_{t}=x\right)}{p\left(X_{t+\tau }^{j}=x^{\prime j}\;|\;X_{t}^{j}=x^{j},X_{t}^{\left[ij\right]}=x^{\left[ij\right]}\right)}\right) \end{array} \end{array}$$
2. For **continuous, linear-Gaussian variables**:
  $$\begin{array}{c} TE_{\tau }\left(X^{i}\to X^{j}\;|\;X^{\left[ij\right]}\right)=\frac{1}{2}\log\left(\frac{\det\Sigma \left(X_{t+\tau }^{j}\;|\;X_{t}^{j}⊕X_{t}^{\left[ij\right]}\right)}{\det\Sigma \left(X_{t+\tau }^{j}\;|\;X_{t}\right)}\right) \end{array}$$
3. For **continuous variables** with an arbitrary distribution, we must resort to the nearest-neighbour methods introduced by [24]. See reference for details.

#### 2.2.9. Other Measures

As already mentioned, all the measures reviewed here (besides CD) were inspired by the $\Phi_{2008}$ measure, which arose from the version of IIT laid out in Ref. [12]. The most recent version of IIT [9] is conceptually distinct, and the associated “Φ-3.0” is consequently different to the measures we consider here. The consideration of perturbation of the system, as well as all of its subsets, in both the past and the future renders Φ-3.0 considerably more computationally expensive than other Φ measures. We do not here attempt to consider the construction of an analogue of Φ-3.0 for spontaneous information dynamics. Such an undertaking lies beyond the scope of this paper.

Recently, Tegmark [17] developed a comprehensive taxonomy of all integrated information measures that can be written as a distance between a probability distribution pertaining to the whole and one obtained as a product of probability distributions pertaining to the parts. Tegmark further identified a shortlist of candidate measures, based on a set of explicit desiderata. This shortlist overlaps with the measures we consider here, and also contains other measures which are minor variants. Of Tegmark’s shortlisted measures, $\phi^{M}$ is equivalent to $\tilde{\Phi }$ under the system’s spontaneous distribution, $\phi_{kk^{\prime }}^{M}$ is its state-resolved version, $\phi^{\mathrm{oak}}$ is transfer entropy (which we cover here through CD), and $\phi^{\mathrm{npk}}$ is not defined for continuous variables. The measures $\Phi_{G}$ and ψ are outside of Tegmark’s classification scheme.

## 3. Results

All of the measures of integrated information that we have described have the potential to behave in ways which are not obvious a priori, and in a manner difficult to express analytically. While some simulations of Φ, $\tilde{\Phi }$ and CD on networks have been performed [2,13], and some analytical understanding has been achieved for Φ and $\tilde{\Phi }$ [5,17], $\Phi^{*}$ and $\Phi_{G}$ have not previously been computed on models consisting of more than two components, and ψ hasn’t previously been explored at all on systems with continuous variables. In this section, we study all the measures together on small networks. We compare the behaviour of the measures, and assess the extent to which each measure reflects different forms of integration and segregation as characterised via correlations and connectivity matrices.

To recap, we consider the following six measures:

- Whole-minus-sum integrated information, Φ,
- Integrated stochastic interaction, $\tilde{\Phi }$,
- Decoder-based integrated information, $\Phi^{*}$,
- Geometric integrated information, $\Phi_{G}$,
- Integrated synergy, ψ,
- Causal density, CD.

We use models based on stochastic linear auto-regressive (AR) processes with Gaussian variables. These constitute appropriate models for testing the measures of integrated information. They are straightforward to parameterise and simulate, and are amenable to the formulae presented in the previous section. Mathematically, we define an AR process (of order 1) by the update equation

(32) $$X_{t+1}=AX_{t}+\varepsilon_{t},$$

where $\varepsilon_{t}$ is a serially independent random sample from a zero-mean Gaussian distribution with given covariance Σ(ε), usually referred to as the *noise* or *error term*. A particular AR process is completely specified by the coupling matrix or *network* *A* and the noise covariance matrix Σ(ε). An AR process is stable, and stationary, if the spectral radius of the coupling matrix is less than 1 [48]. (The spectral radius is the largest of the absolute values of its eigenvalues.) All the example systems we consider are calibrated to be stable, so the Φ measures can be computed from their stationary statistics.

We shall consider how the measures vary with respect to: (i) the strength of connections, i.e., the magnitude of non-zero terms in the coupling matrix; (ii) the topology of the network, i.e., the arrangement of the non-zero terms in the coupling matrix; (iii) the density of connections, i.e., the density of non-zero terms in the coupling matrix; and (iv) the correlation between noise inputs to different system components, i.e., the off diagonal terms in Σ(ε). The strength and density of connections can be thought of as reflecting, in different ways, the level of integration in the network. The correlation between noise inputs reflects (inversely) the level of segregation, in some sense. We also, in each case, compute the control measures

- Time-delayed mutual information (TDMI), $I(X_{t-\tau },X_{t})$; and
- Average absolute correlation $\bar{\Sigma }$, defined as the average absolute value of the non-diagonal entries in the system’s correlation matrix.

These simple measures quantify straightforwardly the level of interdependence between elements of the system, across time and space, respectively. TDMI captures the total information generated as the system transitions from one time-step to the next, and $\bar{\Sigma }$ is another basic measure of the level of integration.

We report the unnormalised measures minimised over even-sized bipartitions—i.e., bipartitions in which both parts have the same number of components. In doing this, we avoid conflating the effects of the choice of definition of effective information with those of the choice of partition search (see section on MIP above). See the Discussion for more details on this topic.

### 3.1. Key Quantities for Computing the Integrated Information Measures

To compute the integrated information measures, the stationary covariance and lagged partial covariance matrices are required. By taking the expected value of $X_{t}^{T}X_{t}$ with Equation (32) and given that $\varepsilon_{t}$ is white noise, uncorrelated in time, one obtains that the stationary covariance matrix Σ(X) is given by the solution to the discrete-time Lyapunov equation,

(33) $$\begin{array}{c} \Sigma \left(X_{t}\right)=A\;\Sigma \left(X_{t}\right)\;A^{T}+\Sigma \left(\epsilon_{t}\right). \end{array}$$

This can be easily solved numerically, for example in Matlab (R2017a, MathWorks, Natick, MA, USA) via use of the dlyap command. The lagged covariance can also be calculated from the parameters of the AR process as

(34) $$\begin{array}{c} \Sigma \left(X_{t-1},X_{t}\right)=\langle X_{t}{\left(AX_{t}+\varepsilon_{t}\right)}^{T}\rangle =\Sigma \left(X_{t}\right)A^{T}, \end{array}$$

and partial covariances can be obtained by applying Equation (7). Finally, we obtain the analogous quantities for the partitions by the marginalisation properties of the Gaussian distribution. Given a bipartition $X_{t}=\left\{M_{t},N_{t}\right\}$, we write the covariance and lagged covariance matrices as

(35) $$\begin{array}{c} \begin{array}{c} \Sigma \left(X_{t}\right)=\left(\begin{array}{cc} \Sigma {\left(X_{t}\right)}_{mm} & \Sigma {\left(X_{t}\right)}_{mn}\\ \Sigma {\left(X_{t}\right)}_{nm} & \Sigma {\left(X_{t}\right)}_{nn} \end{array}\right),\\ \Sigma \left(X_{t-1},X_{t}\right)=\left(\begin{array}{cc} \Sigma {\left(X_{t-1},X_{t}\right)}_{mm} & \Sigma {\left(X_{t-1},X_{t}\right)}_{mn}\\ \Sigma {\left(X_{t-1},X_{t}\right)}_{nm} & \Sigma {\left(X_{t-1},X_{t}\right)}_{nn} \end{array}\right), \end{array} \end{array}$$

and we simply read the partition covariance matrices as

(36) $$\begin{array}{c} \begin{array}{c} \Sigma \left(M_{t}\right)=\Sigma {\left(X_{t}\right)}_{mm}\;,\\ \Sigma \left(M_{t-1},M_{t}\right)=\Sigma {\left(X_{t-1},X_{t}\right)}_{mm}\;. \end{array} \end{array}$$

### 3.2. Two-Node Network

We begin with the simplest non-trivial AR process,

(37a) $$\begin{array}{cc} A\; & =\left(\begin{array}{cc} a & a\\ a & a \end{array}\right), \end{array}$$

(37b) $$\begin{array}{cc} \Sigma (\epsilon ) & =\left(\begin{array}{cc} 1 & c\\ c & 1 \end{array}\right). \end{array}$$

**Figure 1:**
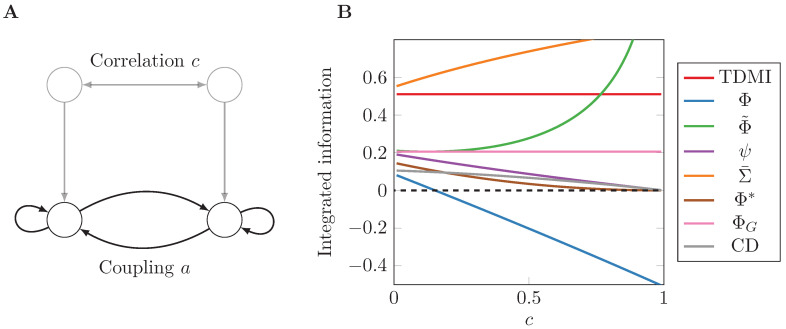
(**A**) graphical representation of the two-node AR process described in Equation (37). Two connected nodes with coupling strength *a* receive noise with correlation *c*, which can be thought of as coming from a common source; (**B**) all integrated information measures for different noise correlation levels *c*.

Setting a=0.4, we obtain the same model as depicted in Figure 3 in Reference [5]. We simulate the AR process with different levels of noise correlation *c* and show results for all the measures in Figure 1. Note that, as *c* approaches 1, the system becomes degenerate, so some matrix determinants in the formulae become zero causing some measures to diverge.

Inspection of Figure 1 immediately reveals a wide variability of behaviour among the measures, in both value and trend, even for this minimally simple model. A good candidate measure of (dynamical) integrated information should tend to 0 as the noise tends to becoming perfectly correlated (c→1) because, in that instance, the whole just becomes a collection of copies of the parts (we don’t consider c=1 because the Gaussian model becomes singular in this limit). Only the measures ψ, $\Phi^{*}$, and CD achieve this. $\Phi_{G}$ is here unaffected by noise correlation [49], and $\tilde{\Phi }$ grows monotonically with *c*. Furthermore, $\tilde{\Phi }$ diverges to infinity as c→1. On the other hand, Φ also decreases monotonically but becomes negative for large enough *c*.

**Figure 2:**
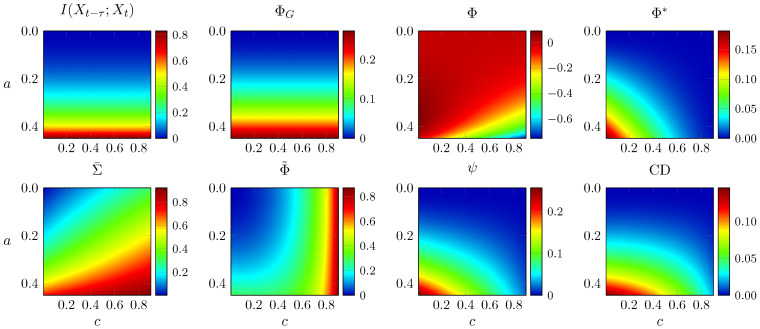
All integrated information measures for the two-node AR process described in Equation (37), for different coupling strengths *a* and noise correlation levels *c*. The vertical axis is inverted for visualisation purposes.

In Figure 2, we analyse the same system, but now varying both noise correlation *c* and coupling strength *a*. As per the stability condition presented above, any value of a≥0.5 makes the system’s spectral radius greater than or equal to 1, so the system becomes non-stationary and variances diverge. Hence, in these plots, we evaluate all measures for values of *a* below the limit a=0.5.

Again, the measures behave very differently. In this case, TDMI and $\Phi_{G}$ remain unaffected by noise correlation, and grow with increasing coupling strength as expected. In contrast, $\tilde{\Phi }$ and $\bar{\Sigma }$ increase with both *a* and *c*. Φ decreases with *c* but shows non-monotonic behaviour with *a*. Three of the measures, ψ, $\Phi^{*}$, and CD, show properties consistent with capturing conjoined segregation and integration—they monotonically decrease with noise correlation and increase with coupling strength.

### 3.3. Eight-Node Networks

We now turn to networks with eight nodes, enabling examination of a richer space of dynamics and topologies.

**Figure 3:**
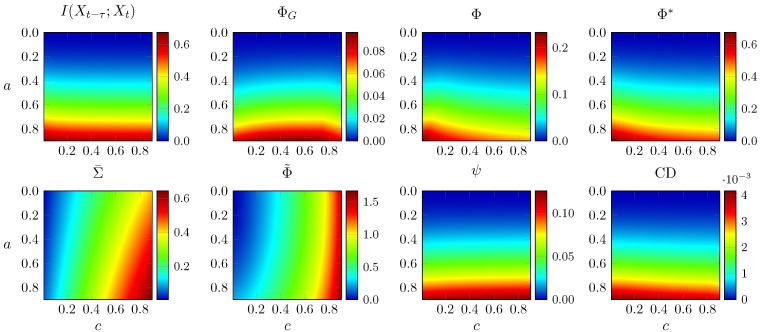
All integrated information measures for the Φ-optimal AR process proposed by [2], for different coupling strengths *a* and noise correlation levels *c*. Vertical axis is inverted for visualisation purposes.

We first analyse a weighted network optimised using a genetic algorithm to yield high Φ (Figure 2b in [2]). The noise covariance matrix has ones in the diagonal and *c* everywhere else, and now *a* is a global factor applied to all edges of the network. The (weighted) adjacency matrix is scaled such that its spectral radius is 1 when a=1. Similar to the previous section, we evaluate all measures for multiple values of *a* and *c* and show the results in Figure 3.

Moving to a larger network mostly preserves the features highlighted above. TDMI is unaffected by *c*; $\tilde{\Phi }$ behaves like $\bar{\Sigma }$ and diverges for large *c*; and $\Phi^{*}$ and CD have the same trend as before, although now the decrease with *c* is less pronounced. Interestingly, ψ and $\Phi_{G}$ increase slightly with *c*, and Φ does not show the instability and negative values seen in Figure 2. Overall, in this more complex network, the effect of increasing noise correlation on Φ, ψ, $\Phi^{*}$, and CD is not as pronounced as in simpler networks, where these measures decrease rapidly towards zero with increasing *c*.

Thus far, we have studied the effect of AR dynamics on integrated information measures, keeping the topology of the network fixed and changing only global parameters. We next examine the effect of network topology, on a set of six networks:

**A**  A fully connected network without self-loops.
**B**  The Φ-optimal binary network presented in [2].
**C**  The Φ-optimal weighted network presented in [2].
**D**  A bidirectional ring network.
**E**  A “small-world” network, formed by introducing two long-range connections to a bidirectional ring network.
**F**  A unidirectional ring network.

**Figure 4:**
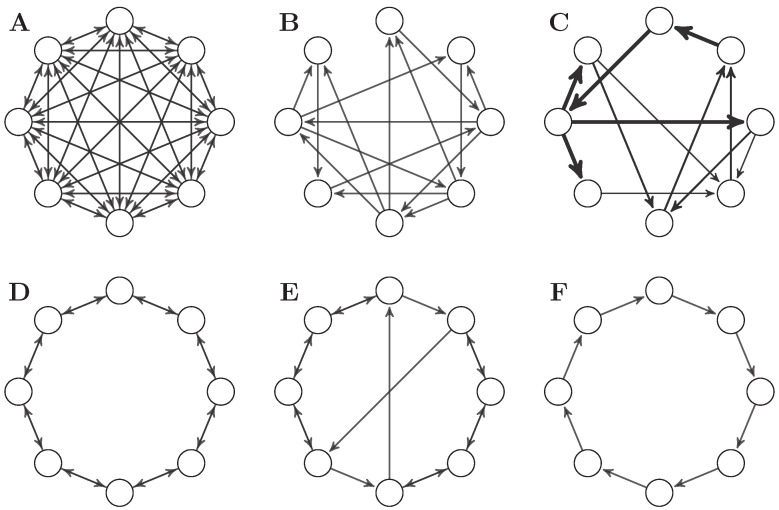
Networks used in the comparative analysis of integrated information measures. (**A**) fully connected network; (**B**) Φ-optimal binary network from [2]; (**C**) Φ-optimal weighted network from [2]; (**D**) bidirectional ring network; (**E**) small world network; and (**F**) is a unidirectional ring network.

**Figure 5:**
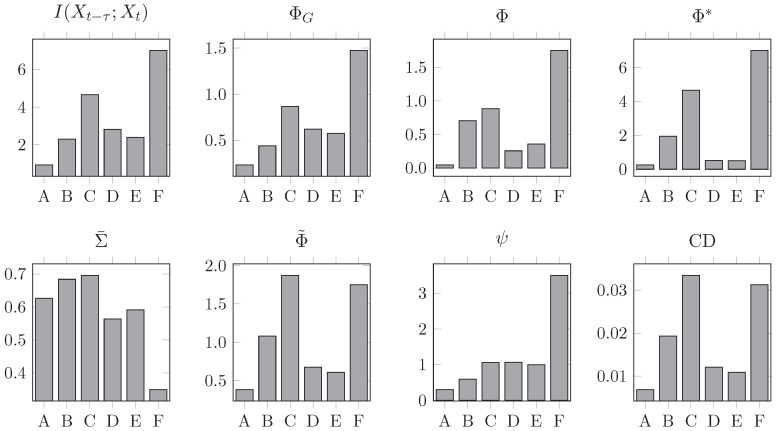
Integrated information measures for all networks in the suite shown in Figure 4, normalised to spectral radius 0.9 and under the influence of uncorrelated noise. The ring and weighted Φ-optimal networks score consistently at the top, while denser networks like the fully connected and the binary Φ-optimal networks are usually at the bottom. Most measures disagree on specific values but agree on the relative ranking of the networks.

In each network, the adjacency matrix has been normalised to a spectral radius of 0.9. As before, we simulate the system following Equation (32), and here set noise input correlations to zero (c=0) so the noise input covariance matrix is just the identity matrix. Figure 4 shows connectivity diagrams of the networks for visual comparison, and Figure 5 shows the values of all integrated information measures evaluated on all networks.

As before, there is substantial variability in the behaviour of all measures, but some general patterns are apparent. Intriguingly, the unidirectional ring network consistently scores highest for all measures (except for $\tilde{\Phi }$ and CD), followed in most cases by the weighted Φ-optimal network [50]. On the other end of the spectrum, the fully connected network **A** consistently scores lowest, which is explained by the large correlation between its nodes as shown by $\bar{\Sigma }$.

The results here can be summarised by comparing the rank assigned to the networks by each measure (see Table 3). Inspecting this table reveals a remarkable alignment between TDMI, $\Phi_{G}$, $\Phi^{*}$, and ψ, especially given how much their behaviour diverges when varying *a* and *c*. Although the particular values are different, the measures largely agree on the ranking of the networks based on their integrated information. This consistency of ranking is initially encouraging with regard to empirical application.

However, the ranking is not what might be expected from topological complexity measures from network theory. If we ranked these networks by e.g., small-world index (SWI) [51,52,53], we expect networks **B**, **C**, and **E** to be at the top and networks **A**, **D**, and **F** to be at the bottom—very different from any of the rankings in Table 3. In fact, the Spearman correlation between the ranking by small-world index and those by TDMI, $\Phi_{G}$, $\Phi^{*}$, and ψ is around −0.4, leading to the conclusion that more structurally complex networks integrate *less* information. We note that these rankings are very robust to noise correlation (results not shown) for all measures except Φ. Across all simulations in this study, the behaviour of Φ is erratic, undermining prospects for empirical application. (This behaviour is even more prevalent if Φ is optimised over all bipartitions, as opposed to over even bipartitions.)

### 3.4. Random Networks

We next perform a more general analysis of the performance of measures of integrated information, using Erdős–Rényi random networks. We consider Erdős–Rényi random networks parametrised by two numbers: the edge density of the network ρ and the noise correlation *c* (defined as above), both in the [0,1) interval. To sample a network with a given ρ, we generate a matrix in which each possible edge is present with probability ρ and then remove self-loops. The stochasticity in the construction of the Erdős–Rényi network induces fluctuations on the integrated information measures, such that, for each (ρ,c), we calculate the mean and variance of each measure.

**Figure 6:**
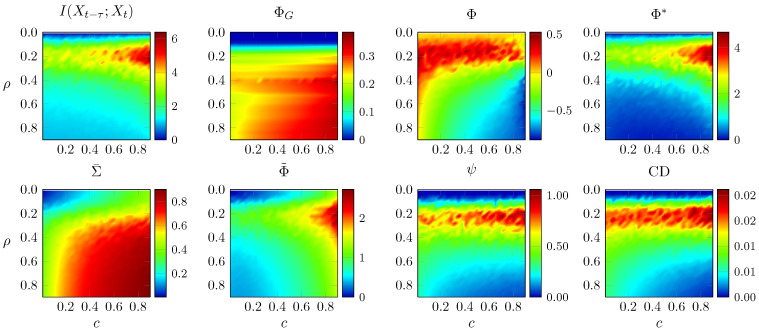
Average integrated information measures for Erdős–Rényi random networks with given density ρ and noise correlation *c*. The vertical axis is inverted for consistency with Figure 2 and Figure 3.

First, we generate 50 networks for each point in the (ρ,c) plane and take the mean of each integrated information measure evaluated on those 50 networks. As before, the adjacency matrices are normalised to a spectral radius of 0.9. Results are shown in Figure 6.

$\Phi_{G}$ increases markedly with ρ and moderately with *c*, $\bar{\Sigma }$ increases sharply with both and the rest of the measures can be divided in two groups, with Φ, ψ and CD that decrease with *c* and TDMI, $\tilde{\Phi }$ and $\Phi^{*}$ that increase. Notably, all integrated information measures except $\Phi_{G}$ show a band of high value at an intermediate value of ρ. This demonstrates their sensitivity to the level of integration. The decrease when ρ is increased beyond a certain point is due to the weakening of the individual connections in that case (due to the fixed overall coupling strength, as quantified by spectral radius).

**Figure 7:**
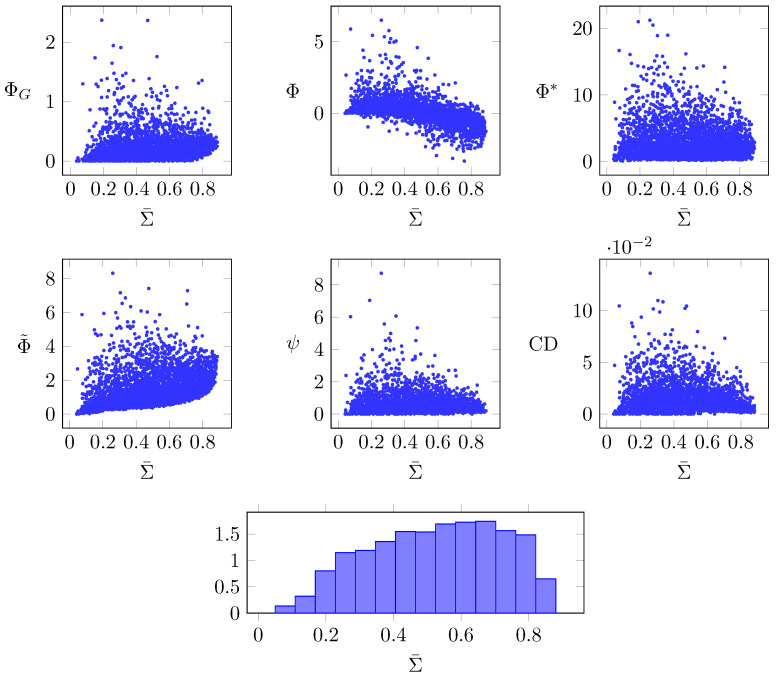
Integrated information measures of random Erdős–Rényi networks, plotted against the average correlation $\bar{\Sigma }$ of the same network; (bottom) normalised histogram of $\bar{\Sigma }$ for all sampled networks.

Secondly, in Figure 7, we plot each measure against the average correlation of each network, following the rationale that dynamical complexity should (as a necessary, but not sufficient condition) peak at an intermediate value of $\bar{\Sigma }$—i.e., it should reach its maximum value in the middle range of $\bar{\Sigma }$. To obtain this figure, we sampled a large number of Erdős–Rényi networks with random (ρ,c), and evaluated all integrated information measures, as well as their average correlation $\bar{\Sigma }$.

Figure 7 shows that some of the measures have this intermediate peak, in particular: $\Phi^{*}$, ψ, $\Phi_{G}$, and CD. Although also showing a modest intermediate peak, $\tilde{\Phi }$ has a stronger overall positive trend with $\bar{\Sigma }$, and Φ an overall negative trend. These analyses further support the notion that $\Phi^{*}$, ψ, $\Phi_{G}$, and CD reflect some form of dynamical complexity, although the relation between them remains unclear and not always consistent in other scenarios.

One might worry that these peaks could be due to a biased sampling of the $\bar{\Sigma }$ axis—if our sampling scheme were obtaining many more samples in, say, the $0.2<\bar{\Sigma }<0.4$ range, then the points with high Φ we see in that range could be explained by the fact that the high-Φ tails of the distribution are sampled better in that range than in the rest of the $\bar{\Sigma }$ axis. However, the histogram at the bottom of Figure 7 shows this is not the case—on the contrary, the samples are relatively uniformly spread along the axis. Therefore, the peaks shown by $\Phi^{*}$, ψ, $\Phi_{G}$, and CD are not sampling artefacts.

## 4. Discussion

In this study, we compared several candidate measures of integrated information in terms of their theoretical construction, and their behaviour when applied to the dynamics generated by a range of non-trivial network architectures. We found that no two measures had precisely the same basic mathematical properties (see Table 2). Empirically, we found a striking variability in the behaviour among the measures even for simple systems; see Table 4 for a summary. Three of the measures, ψ, $\Phi^{*}$ and CD, capture conjoined segregation and integration on small networks, when animated with Gaussian linear AR dynamics (Figure 1). These measures decrease with increasing noise input correlation and increase with increasing coupling strength (Figure 3). Furthermore, on random networks with fixed overall coupling strength (as quantified by spectral radius), they achieve their highest scores when an intermediate number of connections are present (Figure 6). They also obtain their highest scores when the average correlation across components takes an intermediate value (Figure 7).

In terms of network topology, none of the measures strongly reflect complexity of the network structure in a graph theoretic sense. At fixed overall coupling strength, a simple ring structure (Figure 4) leads in most cases to the highest scores. Among the other measures: $\tilde{\Phi }$ is largely determined by the level of correlation amongst the noise inputs, and is not very sensitive to changes in coupling strength; $\Phi_{G}$ depends mainly on the overall coupling strength, and is not very sensitive to changes in noise input correlation; and Φ generally behaves erratically.

Considered together, our results motivate the continued development of ψ, $\Phi^{*}$ and CD as theoretically sound and empirically adequate measures of dynamical complexity.

### 4.1. Partition Selection

Integrated information is typically defined as the effective information beyond the minimum information partition [12,54]. However, when a particular measure of integrated information has been first introduced, it is often with a new operationalisation of both effective information and the minimum information partition. In this paper, we have restricted attention to comparing different choices of measure of effective information, while keeping the same partition selection scheme across all measures. Specifically, we restricted the partition search to even-sized bipartitions, which has the advantage of obviating the need for introducing a normalisation factor when comparing bipartitions with different sizes. For uneven partitions, normalisation factors are required to compensate for the fact that there is less capacity for information sharing as compared to even partitions. However, such factors are known to introduce instabilities, both under continuous parameter changes, and in terms of numerical errors [2]. Further research is needed to compare different approaches to defining the minimum information partition, or finding an approximation to it in reasonable computation time [55].

In terms of computation time, performing the most thorough search, through all partitions, as in the early formulation of Φ by Balduzzi and Tononi [12] requires time $O(n^{n})$. Restricting attention to bipartitions reduces this to $O(2^{n})$, whilst restricting to even bipartitions reduces this further to $O(n^{2})$. These observations highlight a trade-off between computation time and comprehensive consideration of possible partitions. Future comparisons of integrated information measures may benefit from more advanced methods for searching among a restricted set of partitions to obtain a good approximation to the minimum information partition. For example, Toker and Sommer use graph modularity, stochastic block models or spectral clustering as informed heuristics to suggest a small number of partitions likely to be close to the MIP, and then take the minimum over those. With these approximations, they are able to calculate the MIP of networks with hundreds of nodes [6,55]. Alternatively, Hidaka and Oizumi make use of the submodularity of mutual information to perform efficient optimisation and find the bipartition across which there is the least instantaneous mutual information of the system [56]. Presently, however, their method is valid only for instantaneous mutual information and is therefore not applicable to finding the bipartition that minimises any form of normalised effective information as described above in the section dedicated to the MIP.

Furthermore, each measure carries special considerations regarding partition search. For example, for ψ, taking the minimum across all partitions is equivalent to taking it across bipartitions only, thanks to the properties of $I_{\cap }$ [23,27,31]. Arsiwalla and Verschure [57] used $\tilde{\Phi }$ and suggested always using the atomic partition on the basis that it is fast, well-defined, and, for $\tilde{\Phi }$ specifically, it can be proven to be the partition of *maximum* information; and thus it provides a quickly computable upper bound for the measure.

### 4.2. Continuous Variables and the Linear Gaussian Assumption

We have compared the various integrated information measures only on systems whose states are given by continuous variables with a Gaussian distribution. This is motivated by measurement variables being best characterised as continuous in many domains of potential application. Future research should continue the comparison of these measures on a test-bed of systems with discrete variables. Moreover, non-Gaussian continuous systems should also be considered because the Gaussian approximation is not always a good fit to real data. For example, the spiking activity of populations of neurons typically exhibit exponentially distributed dynamics [58]. Systems with discrete variables are in principle straightforward to deal with, since calculating probabilities (following the most brute-force approach) amounts simply to counting occurrences of states. General continuous systems, however, are less straightforward. Estimating generic probability densities in a continuous domain is challenging, and calculating information-theoretic quantities on these is difficult [24,59]. The AR systems we have studied here are a rare exception, in the sense that their probability density can be calculated and all relevant information-theoretic quantities have an analytical expression. Nevertheless, the Gaussian assumption is common in biology, and knowing now how these measures behave on these Gaussian systems will inform further development of these measures, and motivate their application more broadly.

### 4.3. Empirical as Opposed to Maximum Entropy Distribution

We have considered versions of each measure that quantify information with respect to the empirical, or spontaneous, stationary distribution for the state of the system. This constitutes a significant divergence from the supposedly fundamental measures of intrinsic integrated information of IIT versions 2 and 3 [9,12]. Those measures are based on information gained about a hypothetical past moment in which the system was equally likely to be in any one of its possible states (the “maximum entropy” distribution). However, as pointed out previously [2], it is not possible to extend those measures, developed for discrete Markovian systems, to continuous systems. This is because there is no uniquely defined maximum entropy distribution for a continuous random variable (unless it has hard-bounds, i.e., a closed and bounded set of states). Hence, quantification of information with respect to the empirical distribution is the pragmatic choice for construction of an integrated information measure applicable to continuous time-series data.

The consideration of information with respect to the empirical, as opposed to maximum entropy, distribution does, however, have an effect on the concept underlying the measure of integrated information—it results in a measure not of mechanism, but of dynamics [60]. That is, what is measured is not information about what the possible mechanistic causes of the current state *could be*, but rather what the likely preceding states *actually are*, on average, statistically; see [2] for further discussion. Given the diversity of behaviour of the various integrated information measures considered here even on small networks with linear dynamics, one must remain cautious about considering them as generalisations or approximations of the proposed “fundamental” Φ measures of IIT versions 2 or 3 [9,12].

A remaining important challenge, in many practical scenarios, is the identification of stationary epochs. For a relatively long data segment, it can be unrealistic to assume that all the statistics are constant throughout. For shorter data segments, one can not be confident that the system has explored all the states that it potentially would have, given enough time.

## 5. Final Remarks

The further development, and empirical application of Integrated Information Theory requires a good understanding of the various potential operational measures of information integration. During the last few years, several measures have been proposed, but their behaviour in any but the simplest cases has not been extensively characterised or compared. In this study, we have reviewed several candidate measures of (dynamical/empirical) integrated information, and provided a comparative analysis on simulated data, generated by simple Gaussian dynamics applied to a range of network topologies.

Assessing the degree of dynamical complexity, integrated information, or co-existing integration and segregation exhibited by a system remains an important outstanding challenge. Progress in meeting this challenge will have implications not only for theories of consciousness, such as Integrated Information Theory, but more generally in situations where relations between local and global dynamics are of interest. The review presented here identifies promising theoretical approaches for designing adequate measures of integrated information. Furthermore, our simulations demonstrate the need for empirical investigation of such measures, since measures that share similar theoretical properties can behave in substantially different ways, even on simple systems.

## Figures and Tables

**Table 1:** Integrated information measures considered and original references.

Measure	Description	Reference
Φ	Information lost after splitting the system	[12]
$\tilde{\Phi }$	Uncertainty gained after splitting the system	[2]
ψ	Synergistic predictive information between parts of the system	[3]
$\Phi^{*}$	Past state decoding accuracy lost after splitting the system	[5]
$\Phi_{G}$	Information-geometric distance to system with disconnected parts	[4]
CD	Average pairwise directed information flow	[13]

**Table 2:** Overview of properties of integrated information measures, proofs in Appendix C.

	Φ	$\tilde{\Phi }$	ψ	$\Phi^{*}$	$\Phi_{G}$	CD
Time-symmetric	✓	✓	✕	?	✓	✕
Non-negative	✕	✓	✓	✓	✓	✓
Invariant to variable rescaling	✓	✕	✓	✓	✓	✓
Upper-bounded by time-delayed mutual information	✓	✕	✓	✓	✓	✓
Known estimators for arbitrary real-valued systems	✓	✓	✕	✕	✕	✓
Closed-form expression in discrete and Gaussian systems	✓	✓	✓	✕	✕	✓

**Table 3:** Networks ranked according to their value of each integrated information measure (highest value to the left). We add small-world index as a dynamics-agnostic measure of network complexity.

Measure	Ranking					
$I(X_{t},X_{t+\tau })$	F	C	D	E	B	A
$\Phi_{G}$	F	C	D	E	B	A
Φ	F	C	B	E	D	A
$\Phi^{*}$	F	C	B	E	D	A
$\bar{\Sigma }$	C	B	A	E	D	F
$\tilde{\Phi }$	C	F	B	D	E	A
ψ	F	C	D	E	B	A
CD	C	F	B	D	E	A
SWI	C	E	B	A	D	F

**Table 4:** Integrated information measures considered and brief summary of our results.

Measure	Summary of Results
Φ	Erratic behaviour, negative when nodes are strongly correlated.
$\tilde{\Phi }$	Mostly reflects noise input correlation, not sensitive to changes in coupling.
ψ	Reflects both segregation and integration.
$\Phi^{*}$	Reflects both segregation and integration.
$\Phi_{G}$	Mostly reflects changes in coupling, not sensitive to noise input correlation.
CD	Reflects both segregation and integration.

## Appendix A. Derivation and Concavity Proof of *I* *

### Appendix A.1. Derivation of I * in Gaussian Systems

Here, we provide a closed-form expression for the mismatched decoding information in a Gaussian dynamical system. For clarity, we omit the X,τ,P arguments of $\tilde{I}$ and write it as a function of β only. The formula for $\tilde{I}\left(\beta \right)$ for a stationary continuous random process is

(A1) $$\tilde{I}\left(\beta \right)=-\int dx\;p\left(x\right)\log\int d\tilde{x}\;p\left(\tilde{x}\right)q{\left(x|\tilde{x}\right)}^{\beta }+\int d\tilde{x}\int dx\;p\left(x,\tilde{x}\right)\log q{\left(x|\tilde{x}\right)}^{\beta },\;$$

where p(x) is the distribution for $X_{t}$, $p(x,\tilde{x})$ is the joint distribution for $\left(X_{t},X_{t-\tau }\right)$, and $q(x|\tilde{x})$ is the conditional distribution for $X_{t}$ given $X_{t-\tau }$ under the partitioning in question. The function $\tilde{I}\left(\beta \right)$ also depends on $X_{t}$, τ and P, but for the sake of clarity we omit all arguments except for β, which is the parameter of interest here. When $X_{t}$ is Gaussian with covariance matrix $\Sigma_{X}$ (and mean 0 without loss of generality), we have

(A2) $$p\left(x\right)={\left(2\pi \right)}^{-n/2}{\left|\Sigma_{X}\right|}^{-1/2}\exp\left[-\frac{1}{2}\psi \left(x,\Sigma_{X}^{-1}\right)\right],\;$$

where we define

(A3) $$\psi \left(x,M\right)=:x^{T}Mx$$

for a vector *x* and a matrix *M*. Furthermore,

(A4) $$\begin{array}{ccc} q(x|\tilde{x}) & = & {\left(2\pi \right)}^{-n/2}{\left|\Pi_{X|\tilde{X}}\right|}^{-1/2}\exp\left[-\frac{1}{2}\psi \left(x-\Pi_{X\tilde{X}}\Pi_{X}^{-1}\tilde{x},\Pi_{X|\tilde{X}}^{-1}\right)\right]\;, \end{array}$$

where $\Pi_{X}$ is the block diagonal covariance matrix for $X_{t}$ under the partition, $\Pi_{X\tilde{X}}=:\Sigma_{q}\left(X_{t},X_{t-\tau }\right)=\Pi_{\tilde{X}X}^{T}$ is the block diagonal auto-covariance matrix associated with the partition, and $\Pi_{X|\tilde{X}}$ is the partial covariance

(A5) $$\Pi_{X|\tilde{X}}=\Pi_{X}-\Pi_{X\tilde{X}}\Pi_{X}^{-1}\Pi_{\tilde{X}X}\;.$$

We start with the integral

(A6) $$\int d\tilde{x}\;p\left(\tilde{x}\right)q{\left(x|\tilde{x}\right)}^{\beta }={\left(2\pi \right)}^{-n\beta /2}{\left|\Pi_{X|\tilde{X}}\right|}^{-\beta /2}{\left(2\pi \right)}^{-n/2}{\left|\Sigma_{X}\right|}^{-1/2}\int d\tilde{x}\;\exp\left(E\right)\;,$$

where

(A7) $$E=\frac{1}{2}{\tilde{x}}^{T}\Sigma_{X}^{-1}\tilde{x}-\frac{\beta }{2}{\tilde{x}}^{T}\Pi_{X}^{-1}\Pi_{\tilde{X}X}\Pi_{X|\tilde{X}}^{-1}\Pi_{X\tilde{X}}\Pi_{X}^{-1}\tilde{x}+\beta x^{T}\Pi_{X|\tilde{X}}^{-1}\Pi_{X\tilde{X}}\Pi_{X}^{-1}\tilde{x}-\frac{\beta }{2}x^{T}\Pi_{X|\tilde{X}}^{-1}x\;.$$

If we write

(A8) $$E=-\frac{1}{2}{\left(\tilde{x}-Bx\right)}^{T}Q\left(\tilde{x}-Bx\right)-\frac{1}{2}x^{T}R_{1}x\;,$$

then

(A9a) $$\begin{array}{cc} Q & =\Sigma_{X}^{-1}+\beta \Pi_{X}^{-1}\Pi_{\tilde{X}X}\Pi_{X|\tilde{X}}^{-1}\Pi_{X\tilde{X}}\Pi_{X}^{-1}\;, \end{array}$$

(A9b) $$\begin{array}{cc} B^{T} & =\beta \Pi_{X|\tilde{X}}^{-1}\Pi_{X\tilde{X}}\Pi_{X}^{-1}Q^{-1}\;, \end{array}$$

(A9c) $$\begin{array}{cc} R_{1} & =\beta \Pi_{X|\tilde{X}}^{-1}-\beta^{2}\Pi_{X|\tilde{X}}^{-1}\Pi_{X\tilde{X}}\Pi_{X}^{-1}Q^{-1}\Pi_{X}^{-1}\Pi_{\tilde{X}X}\Pi_{X|\tilde{X}}^{-1}\;, \end{array}$$

so

(A10) $$\begin{array}{c} \begin{array}{cc} \int d\tilde{x}\;\exp\left(E\right) & =\exp\left(-\frac{1}{2}x^{T}R_{1}x\right)\int dy\;\exp\left(-\frac{1}{2}y^{T}Qy\right)\\ & =\exp\left(-\frac{1}{2}x^{T}R_{1}x\right){\left(2\pi \right)}^{n/2}{\left|Q\right|}^{-1/2}\;. \end{array} \end{array}$$

Hence, using Equations (A2) and (A6), we obtain the first term in Equation (A1):

(A11) $$\begin{array}{c} -\int dx\;p\left(x\right)\log\int d\tilde{x}\;p\left(\tilde{x}\right)q{\left(x|\tilde{x}\right)}^{\beta }=\frac{n\beta }{2}\log2\pi +\frac{1}{2}\log\left(\left|Q|\cdot \right|\Sigma_{X}\left|\cdot \right|\Pi_{X|\tilde{X}}{|}^{\beta }\right)+\frac{1}{2}\mathrm{tr}\left(\Sigma_{X}R_{1}\right)\;. \end{array}$$

Now, moving on to the second term in Equation (A1),

(A12) $$\int d\tilde{x}\int dx\;p\left(x,\tilde{x}\right)\log q{\left(x|\tilde{x}\right)}^{\beta }=-\frac{\beta n}{2}\log2\pi -\frac{\beta }{2}\log\left|\Pi_{X|\tilde{X}}\right|-\frac{\beta }{2}I_{1}\;,$$

where

(A13) $$\begin{array}{ccc} I_{1} & = & \int d\tilde{x}\int dx\;\;p\left(x,\tilde{x}\right)\;\psi \left(x-\Pi_{X\tilde{X}}\Pi_{X}^{-1}\tilde{x},\Pi_{X|\tilde{X}}^{-1}\right)\\ & = & \int dx\;p\left(x\right)\;\psi \left(x,\Pi_{X|\tilde{X}}^{-1}\right)+\int d\tilde{x}\;p\left(\tilde{x}\right)\;\psi \left(\tilde{x},\Pi_{X}^{-1}\Pi_{\tilde{X}X}\Pi_{X|\tilde{X}}^{-1}\Pi_{X\tilde{X}}\Pi_{X}^{-1}\right)\\ & & -2\int d\tilde{x}\int dx\;p\left(x,\tilde{x}\right)\;x^{T}\Pi_{X|\tilde{X}}^{-1}\Pi_{X\tilde{X}}\Pi_{X}^{-1}\tilde{x}\\ & = & \mathrm{tr}\left(\Pi_{X|\tilde{X}}^{-1}\Sigma_{X}\right)+\mathrm{tr}\left(\Pi_{X}^{-1}\Pi_{\tilde{X}X}\Pi_{X|\tilde{X}}^{-1}\Pi_{X\tilde{X}}\Pi_{X}^{-1}\Sigma_{X}\right)-2\;\mathrm{tr}\left(\Pi_{X|\tilde{X}}^{-1}\Pi_{X\tilde{X}}\Pi_{X}^{-1}\Sigma_{\tilde{X}X}\right),\; \end{array}$$

where $\Sigma_{\tilde{X}X}=:\Sigma \left(X_{t-\tau },X_{t}\right)$. Thus, the second term in Equation (A1) is given by

(A14) $$\begin{array}{c} \begin{array}{cc} \int d\tilde{x}\int dx\;p\left(x,\tilde{x}\right)\log q{\left(x|\tilde{x}\right)}^{\beta }= & -\frac{\beta n}{2}\log2\pi -\frac{\beta }{2}\log\left|\Pi_{X|\tilde{X}}\right|\\ & +\frac{1}{2}\mathrm{tr}\left(\Sigma_{X}R_{2}\right)+\beta \mathrm{tr}\left(\Pi_{X|\tilde{X}}^{-1}\Pi_{X\tilde{X}}\Pi_{X}^{-1}\Sigma_{\tilde{X}X}\right),\; \end{array} \end{array}$$

where

(A15) $$R_{2}=-\beta \Pi_{X|\tilde{X}}^{-1}-\beta \Pi_{X}^{-1}\Pi_{\tilde{X}X}\Pi_{X|\tilde{X}}^{-1}\Pi_{X\tilde{X}}\Pi_{X}^{-1}\;.$$

Finally, putting all the terms in Equations (A11) and (A14) together, we obtain

(A16) $$\tilde{I}\left(\beta \right)=\frac{1}{2}\log\left(\left|Q|\cdot \right|\Sigma_{X}|\right)+\frac{1}{2}\mathrm{tr}\left(\Sigma_{X}R\right)+\beta \;\mathrm{tr}\left(\Pi_{X|\tilde{X}}^{-1}\Pi_{X\tilde{X}}\Pi_{X}^{-1}\Sigma_{\tilde{X}X}\right)\;,$$

where

(A17) $$\begin{array}{ccc} Q & = & \Sigma_{X}^{-1}+\beta \Pi_{X}^{-1}\Pi_{\tilde{X}X}\Pi_{X|\tilde{X}}^{-1}\Pi_{X\tilde{X}}\Pi_{X}^{-1}\;, \end{array}$$

(A18) $$\begin{array}{ccc} R & = & -\beta \Pi_{X}^{-1}\Pi_{\tilde{X}X}\Pi_{X|\tilde{X}}^{-1}\Pi_{X\tilde{X}}\Pi_{X}^{-1}-\beta^{2}\Pi_{X|\tilde{X}}^{-1}\Pi_{X\tilde{X}}\Pi_{X}^{-1}Q^{-1}\Pi_{X}^{-1}\Pi_{\tilde{X}X}\Pi_{X|\tilde{X}}^{-1}\;. \end{array}$$

We note that this formula for $\tilde{I}\left(\beta \right)$ has been verified with numerical methods, and it is not the same as the formula reported by Oizumi et al. [5].

### Appendix A.2. $\tilde{I}$(β) Is Concave in β in Gaussian Systems

Throughout this proof, we will rely multiple times on the the book *Convex Optimization* by Boyd and Vandenberghe [40]. Our aim is to show that $\tilde{I}\left(\beta \right)$ is concave in β, which means it has a unique maximum and can be treated with standard convex optimisation tools. Throughout this proof, we follow Boyd and Vandenberghe’s notation: a function *f* is said to be convex, convex downwards or concave upwards if f(ax+by)≤af(x)+bf(y), for all real non-negative a,b with a+b=1.

We start with the second term in Equation (A1),

(A19) $$\int d\tilde{x}\int dx\;p\left(x,\tilde{x}\right)\log q{\left(x|\tilde{x}\right)}^{\beta }=\beta \int d\tilde{x}\int dx\;p\left(x,\tilde{x}\right)\log q\left(x|\tilde{x}\right),\;$$

which is linear in β. Moving to the first term, using Equation (A4) it can be rewritten as

$$\begin{array}{c} -\int dx\;p\left(x\right)\log\left[\int d\tilde{x}\;p\left(\tilde{x}\right)q{\left(x|\tilde{x}\right)}^{\beta }\right]=-\int dx\;p\left(x\right)\left[-\frac{n\beta }{2}\log2\pi -\frac{\beta }{2}\log\left|\Pi_{X|\tilde{X}}\right|\right]\\ -\int dx\;p\left(x\right)\log\left[p\left(\tilde{x}\right)\exp\left(-\beta f(x,\tilde{x})\right)d\tilde{x}\right]\;. \end{array}$$

We see that the only nonlinear term in $\tilde{I}\left(\beta \right)$ is

(A20) $$-\int dx\;p\left(x\right)\log\left[\int d\tilde{x}\;p\left(\tilde{x}\right)\exp(-\beta f\left(x,\tilde{x}\right)\right],$$

where

(A21) $$f\left(x,\tilde{x}\right)=\frac{1}{2}\psi \left(x-\Pi_{X\tilde{X}}\Pi_{X}^{-1}\tilde{x},\Pi_{X|\tilde{X}}^{-1}\right).$$

Now, we draw from two lemmas presented in [40]:

- An affine function preserves concavity, in the sense that a linear combination of convex (concave) functions is also convex (concave).
- A non-negative weighted sum preserves concavity. Since p(x)>0, the outer integral in Equation (A20) preserves concavity,

With these two remarks, we know that, to prove the concavity of $\tilde{I}\left(\beta \right),$ we just need to prove the concavity of

(A22) $$-\log\left[\int d\tilde{x}\;p\left(\tilde{x}\right)\exp\left(-\beta f(x,\tilde{x})\right)\right].$$

This is known as a log-sum-exp function, which, as per Section 3.1.5 of [40], is convex in β. Finally, the minus sign in the last equation flips the convexity and we conclude that $\tilde{I}\left(\beta \right)$ is concave in β.

## Appendix B. Bounds on Causal Density

We now prove that causal density is upper-bounded by time-delayed mutual information, satisfying what other authors have considered a fundamental requirement for a measure of integrated information [4]. As before, we omit the arguments to CD for clarity. We begin by writing down CD in terms of mutual information:

(A23) $$\begin{array}{c} \begin{array}{cc} \mathrm{CD} & =\frac{1}{n(n-1)}\sum_{i\neq j}{\mathrm{TE}}_{\tau }\left(X^{i}\to X^{j}|X^{\left[ij\right]}\right)\\ & =\frac{1}{n(n-1)}\sum_{i\neq j}I\left(X_{t}^{i};X_{t+\tau }^{j}|X_{t}^{\left[i\right]}\right), \end{array} \end{array}$$

where as before $X_{t}^{\left[i\right]}$ represents the set of all variables in $X_{t}$ except $X_{t}^{i}$. We will use the chain rule of mutual information [18],

(A24) $$\begin{array}{c} I(X;Y,Z)=I(X;Z)+I(X;Y|Z). \end{array}$$

Using this chain rule and the non-negativity of mutual information, we can state that $I\left(X_{t}^{i};X_{t+\tau }^{j}|X_{t}^{\left[i\right]}\right)\leq I\left(X_{t};X_{t+\tau }^{j}\right)$, and therefore

(A25) $$\begin{array}{c} \mathrm{CD}\leq \frac{1}{n(n-1)}\sum_{i\neq j}I\left(X_{t};X_{t+\tau }^{i}\right). \end{array}$$

Also by the same chain rule, it is easy to see that $I\left(X_{t};X_{t+\tau }^{i}\right)\leq I\left(X_{t};X_{t+\tau }\right)$. Then,

(A26) $$\begin{array}{c} \mathrm{CD}\leq \frac{1}{n(n-1)}\sum_{i\neq j}I\left(X_{t};X_{t+\tau }\right)\;. \end{array}$$

Given that the sum runs across all n(n−1) pairs, we arrive at our result

(A27) $$\begin{array}{c} \mathrm{CD}\leq I(X_{t};X_{t+\tau }). \end{array}$$

## Appendix C. Properties of Integrated Information Measures

We prove the properties of in Table 2 of the main text. We will make use of the properties of mutual information introduced in Section 2.1, repeated here for convenience:

MI-1 I(X;Y)=I(Y;X),
MI-2 I(X;Y)≥0,
MI-3 I(f(X);g(Y))=I(X;Y) for any injective functions f,g,

### Appendix C.1. Whole-Minus-Sum Integrated Information *Φ*

Time-symmetric Follows from (MI-1).
Non-negative Proof by example. If $X_{t}^{i}=X_{t}^{j}$, we have $\Phi =\left(1-N\right)I\left(X_{t}^{i};X_{t-\tau }^{i}\right)\leq 0$.
Rescaling-invariant Follows from (MI-3) when Balduzzi and Tononi’s [12] normalisation factor is not used.
Bounded by TDMI Follows from (MI-2).

### Appendix C.2. Integrated Stochastic Interaction $\tilde{\Phi }$

Time-symmetric Follows from $H\left(X_{t}|H_{t-\tau }\right)=H\left(X_{t-\tau }|H_{t}\right)$, which can be proved starting from the system temporal joint entropy

$$\begin{array}{cc} H(X_{t},X_{t-\tau }) & =H\left(X_{t}|X_{t-\tau }\right)+H\left(X_{t-\tau }\right)\\ & =H\left(X_{t-\tau },X_{t}\right)=H\left(X_{t-\tau }|X_{t}\right)+H\left(X_{t}\right), \end{array}$$

and using the fact that by the ergodic property $H\left(X_{t}\right)=H\left(X_{t-\tau }\right)$. The same logic applies to all parts of the system:

Non-negative Follows from the fact that $\tilde{\Phi }$ is an M-projection (see Reference [4]).
Rescaling-invariant Follows from the non-invariance of differential entropy [18] (regardless of whether a normalisation factor is used).
Bounded by TDMI Proof by counterexample. In the two-node AR process of the main text $\tilde{\Phi }\to \infty$ as c→1, although TDMI remains finite.

### Appendix C.3. Integrated Synergy ψ

Time-symmetric Proof by counterexample—for the AR system with
  $$\begin{array}{c} A=\left(\begin{array}{cc} a & a\\ 0 & 0 \end{array}\right)\;,\;\Sigma \left(\varepsilon \right)=\left(\begin{array}{cc} 1 & 0\\ 0 & 1 \end{array}\right). \end{array}$$

We have $\psi =\frac{1}{2}\log\left(1+a^{2}\right),$ while, for the time-reversed process, $\psi =\frac{1}{2}\log\left(1+a^{4}\right)$. Note that this proof applies only to the MMI-PID used in this paper and presented in [23].

Non-negative Follows from $I_{\cup }\left(X,Y;Z\right)<I\left(\left\{X,Y\right\};Z\right)$ [27].
Rescaling-invariant Follows from (MI-3) and the fact that $I_{\cap }$ is also invariant (see property (**Eq**) in Section 5 of [3]).
Bounded by TDMI Follows from the non-negativity of $I_{\cup }$ [27].

### Appendix C.4. Decoder-Based Integrated Information *Φ**

Non-negative Follows from $I^{*}\left[X;\tau ,P\right]\leq I\left(X_{t};X_{t-\tau }\right)$, proven in Reference [36].
Rescaling-invariant Assume that the measure is computed on a time series of rescaled data $X_{t}^{r}=X_{t}A$, where *A* is a diagonal matrix with positive real numbers. Then, its covariance is related to the covariance of the original time series as $\Sigma_{X}^{r}=E\left[{X_{t}^{r}}^{T}X_{t}^{r}\right]=E\left[A^{T}X_{t}^{T}X_{t}A\right]=A^{2}\Sigma_{X}$. We can analogously calculate $\Pi_{X},\Pi_{X\tilde{X}},\Pi_{X|\tilde{X}}$ and easily verify that all *A*’s cancel out, proving the invariance.
Bounded by TDMI Follows from $I^{*}\left[X;\tau ,P\right]\geq 0$, proven in Reference [36].

### Appendix C.5. Geometric Integrated Information *Φ*_G_

Time-symmetric Follows from the symmetry in the constraints that define the manifold of restricted models *Q* [4].
Non-negative Follows from the fact that $\Phi_{G}$ is an M-projection [4].
Rescaling-invariant Given a Gaussian distribution *p* with covariance $\Sigma_{p}$, its M-projection in *Q* is another Gaussian with covariance $\Sigma_{q}$. Given a new distribution $p^{\prime }$ formed by rescaling some of the variables in *p*, the M-projection of $p^{\prime }$ is a Gaussian with covariance $A^{2}\Sigma_{q}$ with *A* a diagonal positive matrix (see above), which satisfies $D_{KL}\left(p∥q\right)=D_{KL}\left(p^{\prime }∥q^{\prime }\right)$ and therefore $\Phi_{G}$ is invariant to rescaling.
Bounded by TDMI TDMI can be defined as the M-projection of the full model *p* to a manifold of restricted models $Q^{MI}=\left\{q\;:q\left(X_{t},X_{t-\tau }\right)=q\left(X_{t}\right)q\left(X_{t-\tau }\right)\right\}$ [4]. The bound $\Phi_{G}\leq I\left(X_{t};X_{t-\tau }\right)$ follows from the fact that $Q^{MI}\subset Q$.

### Appendix C.6. Causal Density

Time-symmetric Follows from the non-symmetry of transfer entropy [61].
Non-negative Re-writing CD as a sum of conditional MI terms, follows from (MI-2).
Rescaling-invariant Follows from (MI-3).
Bounded by TDMI Proven in S2 Appendix.
